# Validity of Bioimpedance Spectroscopy in the Assessment of Total Body Water and Body Composition in Wrestlers and Untrained Subjects

**DOI:** 10.3390/ijerph17249433

**Published:** 2020-12-16

**Authors:** Keisuke Shiose, Emi Kondo, Rie Takae, Hiroyuki Sagayama, Keiko Motonaga, Yosuke Yamada, Yoshinari Uehara, Yasuki Higaki, Hideyuki Takahashi, Hiroaki Tanaka

**Affiliations:** 1 Faculty of Education, University of Miyazaki, Miyazaki 889-2192, Japan; 2 Fukuoka University Institute for Physical Activity, Fukuoka 814-0180, Japan; ueharay@fukuoka-u.ac.jp (Y.U.); higaki@fukuoka-u.ac.jp (Y.H.); htanaka@fukuoka-u.ac.jp (H.T.); 3 Japan Institute of Sports Sciences, Tokyo 115-0056, Japan; emi.kondo@jpnsport.go.jp (E.K.); keiko.motonaga@jpnsport.go.jp (K.M.); takahashi.hideyuk.ga@u.tsukuba.ac.jp (H.T.); 4 Faculty of Sports and Health Science, Fukuoka University, Fukuoka 814-0180, Japan; lrietty.tl@gmail.com; 5 Faculty of Health and Sport Sciences, University of Tsukuba, Tsukuba 305-8577, Japan; sagayama.hiroyuki.ka@u.tsukuba.ac.jp; 6 Department of Physical Activity, National Institutes of Biomedical Innovation, Health and Nutrition, Tokyo 162-8636, Japan; yamaday@nibiohn.go.jp

**Keywords:** anthropometry, deuterium, body mass index (BMI)

## Abstract

Bioimpedance spectroscopy (BIS) is an easy tool to assess hydration status and body composition. However, its validity in athletes remains controversial. We investigated the validity of BIS on total body water (TBW) and body composition estimation in Japanese wrestlers and untrained subjects. TBW of 49 young Japanese male subjects (31 untrained, 18 wrestlers) were assessed using the deuterium dilution method (DDM) and BIS. De Lorenzo’s and Moissl’s equations were employed in BIS for TBW estimation. To evaluate body composition, Siri’s 3-compartment model and published TBW/fat-free mass (FFM) ratio were applied in DDM and BIS, respectively. In untrained subjects, DDM and BIS with de Lorenzo’s equation showed consistent TBW estimates, whereas BIS with Moissl’s equation overestimated TBW (*p* < 0.001 vs. DDM). DDM and BIS with de Lorenzo’s equation estimated FFM and percent of fat mass consistently, whereas BIS with Moissl’s equation over-estimated and under-estimated them (*p* < 0.001 vs. DDM). In wrestlers, BIS with de Lorenzo’s and Moissl’s equations assessed TBW similarly with DDM. However, the Bland–Altman analysis revealed a proportional bias for TBW in BIS with de Lorenzo’s equation (r = 0.735, *p* < 0.001). Body composition assessed with BIS using both equations and DDM were not different. In conclusion, BIS with de Lorenzo’s equation accurately estimates the TBW and body composition in untrained subjects, whereas BIS with Moissl’s equation is more valid in wrestlers. Our results demonstrated the usefulness of BIS for assessing TBW and body composition in Japanese male wrestlers.

## 1. Introduction

Bioimpedance spectroscopy (BIS) is used to measure body water content based on Hanai’s mixture theory by measuring the resistance of extracellular and intracellular components [1]. Previous studies have shown that BIS has good accuracy to estimate body water content in patients and healthy subjects with a reference to a “gold-standard” isotope dilution method and that BIS is a better than traditional single-frequency bioimpedance method because it allows for adjustments in the variations of intracellular and extracellular water [2,3,4]. In addition, BIS can be used to assess body composition, since hydration of fat-free mass (FFM) in both athletes and non-athletes is similar to the theoretical value (~73%) [5].

Regarding the estimation algorithm of BIS for body water assessment, de Lorenzo et al. proposed an estimation equation, which involved standard anthropometric coefficients (i.e., geometrical shape of the body (K_B_) and body density (D_b_)) derived from enlisted people [6]. However, using standard anthropometric coefficients can potentially become a source of error, since actual anthropometric values may not be correct in certain types of subjects. This problem has been noted in lean and obese subjects, where total body water (TBW) assessed by BIS was underestimated in the former and overestimated in the latter [7]. To reduce the estimation error due to the incompatibility of anthropometric coefficients, Moissl et al. proposed the correction for coefficients using a body mass index (BMI) [7].

In trained subjects and athletes, it has been reported that BIS with standard anthropometric coefficients could accurately assess body water content [8,9,10]. However, using algorithm applying standard anthropometric coefficients for athletes is questionable because anthropometric characteristics vary between athletes in different sports. For example, wrestlers are presumed to have unique anthropometric characteristics compared with other athletes and untrained subjects, such as their arms being relatively more muscular than their legs [11,12].

In this study, the validity of BIS in measuring TBW and body composition was investigated in Japanese wrestlers and untrained subjects using two estimation equations: de Lorenzo’s equation, which used the standard anthropometric coefficients, and Moissl’s equation, which used BMI-corrected anthropometric coefficients. We hypothesized that de Lorenzo’s equation would be valid in untrained subjects and that it should have an estimation error in wrestlers because of the differences in their anthropometric characteristics. In addition, the estimation error may be reduced by using Moissl’s equation because it is compatible with anthropometric characteristics in wrestlers.

## 2. Materials and Methods

### 2.1. Subjects

Forty-nine subjects participated in this study, which consisted of 31 male untrained subjects and 18 male college wrestlers. Each subject was fully informed of all the risks and discomforts associated with this study before a written informed consent to participate was given. The study procedure was approved by the ethical committee of Fukuoka University (17–10–06, approved on 8 March 2018) and Japan Institute of Sports Sciences (2014 No. 036, approved on 27 October 2014, and 2016 No. 015, approved on 27 May 2016). Most college non-athletes were sedentary or recreationally active but did not perform regular physical training. Athletes belonged to the East Japan Collegiate Wrestling League and participated in international-level, national-level, and regional-level matches.

On the day of the experiment, the subjects arrived at the laboratory in the morning. They were asked not to consume any food or drink for at least 10 h before the experiment. However, they were allowed to drink water. Vigorous physical activities and alcoholic beverages were restricted one day before the experiment. TBW was estimated using the deuterium (D_2_O) dilution method and BIS at the same time. Subsequently, D_b_ was measured using air displacement plethysmograph or underwater weighting, as described previously [5].

### 2.2. D_2_O Dilution Method

The D_2_O dilution method, as described by previous studies [13,14], was used for the estimation of TBW (${\mathrm{TBW}}_{D_{2}O}$). The first urine samples were obtained, and subjects subsequently ingested 0.06 g/kg body mass of D_2_O diluted with 20 times water. The bottle used to administer the solution was then rinsed twice with about 30 mL of water that was also ingested. Within 2 h after D_2_O ingestion, an additional 300 mL of water was ingested. The second and third urine samples were obtained 3 h and 4 h after D_2_O ingestion, respectively. The urine samples were stored at −30 °C for later analysis. D_2_O abundance in the samples was measured using an isotope-ratio mass spectrometer (Hydra 20–20 Stable Isotope Mass Spectrometer, Sercon, Crewe, UK). The average standard deviations of the Hydra 20–20 in the analyses were reported to be 0.7‰ for ^2^H [15]. A hydrogen gas-water equilibration method using platinum catalysts was used to measure the ratio of ^2^H and ^1^H isotopes [16]. The accuracy of the analyses was calibrated and checked by measuring three different working water standards (low, −57.59‰, middle, 490.64‰, and high, 872.40‰ delta V-SMOW unit) within each batch of samples. The ^2^H dilution space was determined by dividing the dose of administered ^2^H. TBW was calculated as ^2^H dilution space divided by 1.041 for the dilution space measured by ^2^H [14]. Test-retest variability (coefficient of variation) for TBW assessment using the isotope dilution method is 0.6% in our laboratory [17].

### 2.3. Bioimpedance Spectroscopy (BIS)

A BIS system (SFB7, ImpediMed, Pinkenba, QLD, Australia) was used to estimate TBW. Two injection electrodes (Red Dot, 3M Health Care, St. Paul, MN, USA) were placed on the right side of the body, on the dorsal surface of the right hand and right foot, proximal to the metacarpophalangeal and metatarsophalangeal joints, respectively. Sensing electrodes were then placed on the right side of the body at the middle of an imaginary line on the dorsum of the wrist, joining the bony prominences of the radius and ulna, and at the middle of the anterior surface of the ankle, on an imaginary line joining the medial and lateral malleoli. The measurements were done in a temperature-controlled room (21–22 °C) with the subjects lying in a supine position for 15 min.

The frequency range of BIS was 3 kHz to 1 MHz. Resistance at infinity high frequency (R_∞_) and at zero frequency (R_0_) were obtained from the Cole plot [18] using analytical software (Bioimp Software, Impedimed, Pinkenba, QLD, Australia), with the analytical setting as described in previous studies [19,20]. The resistance of the extracellular component (R_e_) was equivalent to R_0_, and resistance of the intracellular components (R_i_) was calculated as 1/((1/R_∞_) − (1/R_0_)). TBW was estimated from R_e_ and R_i_ by using two estimation equations proposed by de Lorenzo et al. [6] and Moissl et al. [7].

de Lorenzo’s equation was embedded into the SFB7 system. TBW calculation with de Lorenzo’s equation (TBW_SFB7_) was conducted using the following formula [6].

(1) $${\mathrm{ECW}}_{\mathrm{SFB}7}=k_{\mathrm{ECW},\;\mathrm{SFB}7}{\left(\frac{L^{2}\sqrt{\mathrm{Wt}}}{R_{e}}\right)}^{\frac{2}{3}}$$

(2) $$k_{\mathrm{ECW},\;\mathrm{SFB}7}=\frac{1}{100}{\left(\frac{K_{B}^{2}\rho_{\mathrm{ECW}}^{2}}{D_{b}}\right)}^{\frac{1}{3}}$$

where ECW_SFB7_ is the extracellular water volume (liters), Wt is the body weight (kg), L is the height (cm), and ρ_ECW_ is the ρ of extracellular water (ohm·cm). ρ_ECW_ was determined as 273.9 ohm·cm from the default setting of the analytical software. K_B_ was identified as 4.03 from the National Institute of Advanced Industrial Science and Technology (AIST) anthropometric database of young Japanese adult men [21]. D_b_ was taken at 1.050 g/mL in conformity with a previous study [6] and the default setting of analytical software.

(3) $${\left(1+\frac{{\mathrm{ICW}}_{\;\mathrm{SFB}7}}{{\mathrm{ECW}}_{\;\mathrm{SFB}7}}\right)}^{\frac{5}{2}}=\left(\frac{R_{e}+R_{i}}{R_{e}}\right)\left(1+\frac{k_{\rho }{\mathrm{ICW}}_{\mathrm{SFB}7}}{{\mathrm{ECW}}_{\;\mathrm{SFB}7}}\right)$$

(4) $$k_{\rho }=\frac{\rho_{\mathrm{ICW}}}{\rho_{\mathrm{ECW}}}$$

where ICW_SFB7_ is the intracellular water volume (liters) and ρ_ICW_ is the ρ of intracellular water (ohm·cm). ρ_ICW_ was determined as 937.2 ohm·cm from the default setting of the analytical software.

(5) $${\mathrm{TBW}}_{\mathrm{SFB}7}={\mathrm{ECW}}_{\mathrm{SFB}7}+{\mathrm{ICW}}_{\mathrm{SFB}7}$$

Moissl’s equation applied variable coefficients according to the individual’s BMI. TBW calculation with Moissl’s equation (TBW_SFB7_) was conducted using the following formula [7].

(6) $${\mathrm{ECW}}_{\mathrm{BMI}}=k_{\mathrm{ECW},\;\mathrm{BMI}}{\left(\frac{L^{2}\sqrt{\mathrm{Wt}}}{R_{e}}\right)}^{\frac{2}{3}}$$

where

(7) $$k_{\mathrm{ECW},\;\mathrm{BMI}}=\frac{a}{\mathrm{BMI}}+b$$

(8) $${\mathrm{ICW}}_{\mathrm{BMI}}=k_{\mathrm{ICW},\;\mathrm{BMI}}{\left(\frac{L^{2}\sqrt{\mathrm{Wt}}}{R_{i}}\right)}^{\frac{2}{3}}$$

where

(9) $$k_{\mathrm{ICW},\mathrm{BMI}}=\frac{c}{\mathrm{BMI}}+d$$

(10) $${\mathrm{TBW}}_{\mathrm{BMI}}={\mathrm{ECW}}_{\mathrm{BMI}}+{\mathrm{ICW}}_{\mathrm{BMI}}$$

The equation parameter was determined as a = 0.188, b = 0.2883, c = 5.8758, and d = 0.4194 in accordance with previous studies [7].

### 2.4. Body Composition

From ${\mathrm{TBW}}_{D_{2}O}$, FFM, and fat mass percentage were assessed using Siri’s three-compartment (3C) model with Db (FFM_3C_ and %Fat_3C_) [22]. In the 3C model, FFM was determined by subtracting the fat mass from the body weight, which was the sum of ${\mathrm{TBW}}_{D_{2}O}$ and fat-free dry solid. From TBW_SFB7_ and TBW_BMI_, FFM (FFM_SFB7_ and FFM_BMI_) and fat mass percentage (%Fat_SFB7_ and %Fat_BMI_) were estimated using the previously reported TBW/FFM ratio (0.721 for untrained subjects and 0.723 for wrestlers) [5].

### 2.5. Statistical Analysis

All data were expressed as a mean ± standard deviation (SD). The Shapiro–Wilk test was used to confirm normality. *T*-tests were applied to evaluate the differences between non-athletes and athletes. To investigate the relationship between TBW estimated with BIS and a D_2_O dilution method, linear regression analysis was performed. To assess the accuracy of TBW estimated using BIS, Lin’s concordance correlation coefficient (CCC) was calculated [23]. The CCC (ρc) includes the factor of precision (ρ) and accuracy (Cb), i.e., ρc = ρ × Cb. ρ is Pearson’s correlation coefficient, and Cb is a bias correction factor that measures how far the best-fit line deviates from a line at 45 degrees. Agreement between methods were assessed using Bland-Altman analysis. Differences were considered statistically significant at *p* < 0.05. Statistical calculations were performed using Microsoft Excel 2016 (Microsoft Corp., Redmond, WA, USA) and SPSS version 21.0 software (IBM, Armonk, NY, USA).

## 3. Results

### 3.1. Subject Characteristics

The subjects’ characteristics are presented in Table 1. In untrained subjects, age and height were significantly high (*p* = 0.006 and *p* = 0.002, respectively), and weight, BMI, and Db were significantly small compared to wrestlers (*p* < 0.001, *p* < 0.001, *p* = 0.003, respectively).

### 3.2. Body Resistance, Total Water Content

Body resistance and body water content measured using BIS and the D_2_O dilution technique are presented in Table 2.

TBW, ECW, and ICW assessed using the D_2_O dilution method and BIS in wrestlers were significantly larger than those in untrained subjects (all *p* < 0.001). In untrained subjects, TBW_BMI_ was significantly larger than ${\mathrm{TBW}}_{D_{2}O}$ (*p* < 0.001). However, there were no differences between ${\mathrm{TBW}}_{D_{2}O}$ and TBW_SFB7_ or TBW_BMI_ in wrestlers. 

### 3.3. Body Composition

Body composition estimated from TBW measured using Siri’s 3C model and BIS are presented in Table 3.

FFM_3C_, FFM_SFB7_, and FFM_BMI_ in wrestlers were significantly higher than those in untrained subjects (all *p* < 0.001). %Fat_3C_, %Fat_SFB7_, and %Fat _BMI_ in wrestlers were significantly lower than those in untrained subjects (*p* < 0.001, *p* < 0.001, *p* = 0.046, respectively). In untrained subjects, FFM_BMI_ was significantly higher than FFM_3C_ (*p* < 0.001), and %Fat_BMI_ was significantly lower than %Fat_3C_ (*p* < 0.001). In wrestlers, FFM_SFB7_ and FFM_BMI_ were not different compared to FFM_3C_, and %Fat_SFB7_ and %Fat_BMI_ were not different when compared to %Fat_3C_.

### 3.4. Regression and CCC Analysis

**Figure 1:**
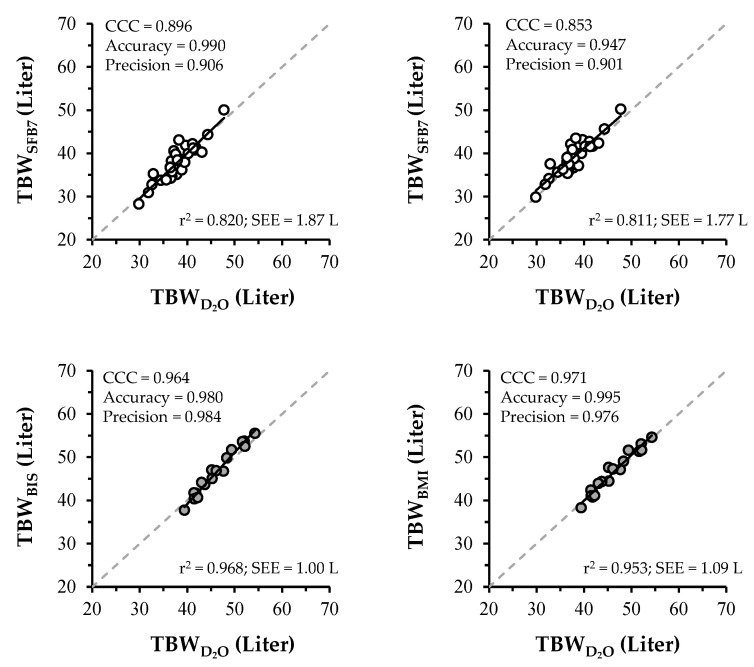
Relationship between ${\mathrm{TBW}}_{D_{2}O}$ and TBW_SFB7_ or TBW_BMI_. ○, non-athletes. ●, athletes. r^2^ and SEE represent coefficient of determination and standard error of estimates, respectively.

Results of regression and CCC analysis are shown in Figure 1. Coefficient of determination (R^2^) for TBW_SFB7_ and TBW_BMI_ were 0.820 and 0.811, respectively, in untrained subjects and were 0.968 and 0.953, respectively, in wrestlers. The standard error of estimate for TBW_SFB7_ and TBW_BMI_ were 1.87 L and 1.77 L, respectively, in untrained subjects and 1.00 L and 1.09 L, respectively, in wrestlers. The CCC values were 0.896 and 0.853 for TBW_SFB7_ and TBW_BMI_, respectively, in untrained subjects and 0.964 and 0.971 for TBW_SFB7_ and TBW_BMI_, respectively, in wrestlers. 

### 3.5. Bland–Altman Analysis

**Figure 2:**
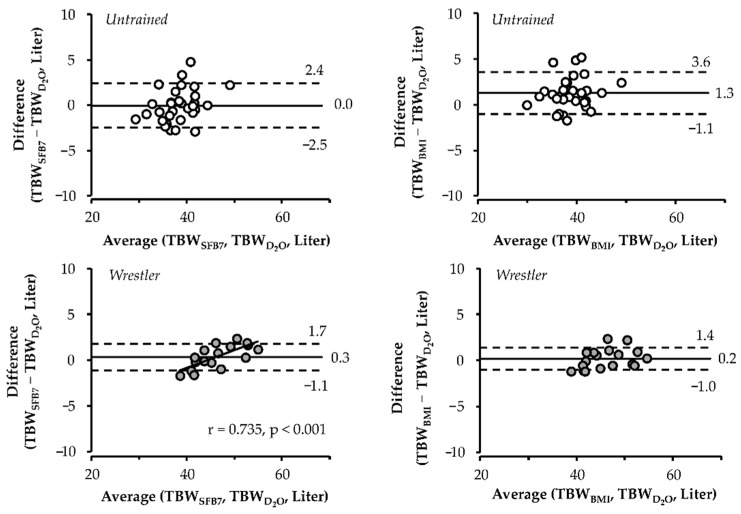
Bland-Altman plot for TBW_SFB7_ and TBW_BMI_ in relation to ${\mathrm{TBW}}_{D_{2}O}$. ○, non-athletes. ●, athletes. The trend line represents mean bias and the regression line between parameters as illustrated by a coefficient of correlation (r). The upper and lower dashed line represents a 95% limit of agreement.

Bland–Altman plot for TBW_SFB7_ and TBW_BMI_ in relation to ${\mathrm{TBW}}_{D_{2}O}$ is shown in Figure 2. In untrained subjects, the mean bias was significantly smaller for TBW_SFB7_ than for TBW_BMI_ (*p* < 0.001). The mean bias for TBW_SFB7_ was 0.0 L (95% CI, −0.7 to 0.6 L) and that for TBW_BMI_ was 1.3 L (95% CI, 0.5 to 2.1 L). The 95% limit of agreement (±1.96 SD) for TBW_SFB7_ was −2.5 to 2.4 L and that for TBW_BMI_ was −1.1 to 3.6 L.

In wrestlers, there was no significant difference in the mean bias between TBW_SFB7_ and TBW_BMI_ (*p* = 0.629). The mean bias for TBW_SFB7_ was 0.3 L (95% CI, −0.3 to 1.0 L) and that for TBW_BMI_ was 0.2 L (95% CI, −0.3 to 0.8 L). The significant correlation between average and difference of ${\mathrm{TBW}}_{D_{2}O}$ and TBW_SFB7_ (r = 0.735, *p* < 0.001) showed proportional bias for TBW_SFB7_, but not for TBW_BMI_. The 95% limit of agreement for TBW_SFB7_ was −1.1 to 1.7 L and that for TBW_BMI_ was −1.0 to 1.4 L.

## 4. Discussion

We evaluated the validity of BIS with de Lorenzo’s and Moissl’s equations against the D_2_O dilution method in Japanese college wrestlers and untrained subjects. BIS with de Lorenzo’s equation accurately estimated TBW and body composition in untrained subjects. However, systematic bias was seen in wrestlers. BIS with Moissl’s equation provided an accurate estimation of the TBW and body composition without systematic bias in wrestlers.

In untrained subjects, no significant difference was found between TBW_SFB7_ and ${\mathrm{TBW}}_{D_{2}O}$. Bland–Altman analysis showed a small mean bias (=0.0 L) and no systematic bias for TBW_SFB7_. The mean bias and limit of agreement of TBW_SFB7_ in untrained subjects was better than those in a previous study [24]. Our results primarily indicate that BIS with de Lorenzo’s equation can correctly assess TBW in untrained Japanese subjects. In calculating for TBW_SFB7_, we used K_B_ = 4.03 and D_b_ = 1.050. K_B_ is determined using only the length and circumference of each body segment (arm, trunk, and leg), and numerous studies have used K_B_ = 4.3, which is determined from anthropometric values in the enlisted Caucasian [6,25]. However, K_B_ of 4.3 is not applicable to Japanese subjects because the length of their anthropometric features and arm/trunk/leg circumference ratio are different from those of Caucasians. Japanese subjects have relatively shorter limbs and smaller trunk circumferences [26]. If 4.3 was used instead of 4.03, the TBW_SFB7_ would have been overestimated by about 1.5 L by our trial calculation. Our data showed that the actual D_b_ was 1.065 g/mL (range, 1.049–1.083 g/mL, Table 1) in untrained subjects, which is 1.5% higher than the proposed value. However, we calculated that this difference was small as it only had a negligible effect on body water estimation (less than 100 mL). Incidentally, we confirm that the use of measured D_b_ instead of the proposed D_b_ did not improve the bias between TBW_SFB7_ and TBW_D2O_. Therefore, the values of K_B_ and D_b_ used in this study were appropriate for Japanese untrained male subjects and helped in improving the validity of TBW_SFB7_.

In contrast to TBW_SFB7_, TBW_BMI_ overestimated ${\mathrm{TBW}}_{D_{2}O}$ by more than 1.0 L in untrained subjects. This error was directly implicated in the estimation error in body composition. Overestimation of TBW_BMI_ was likely related to differences in anthropometric characteristics between races. Originally, correction formula of the TBW_BMI_ was determined to fit the body water content of Caucasians [7]. However, Caucasians have a larger muscle mass, which results in more intracellular water, than Asians, even though their BMI were similar [26,27]. Therefore, relatively small muscle mass in Japanese untrained subjects more than initially assumed should cause overestimation of TBW_BMI_ in this study.

To the best of our knowledge, this is first study demonstrating the validity of BIS with de Lorenzo’s and Moissl’s equations in assessing TBW in wrestlers. TBW_SFB7_ and TBW_BMI_ were equivalent, and both were not different from ${\mathrm{TBW}}_{D_{2}O}$. However, the Bland–Altman analysis revealed a proportional bias for TBW_SFB7_. This result partly contradicts previous results showing no systematic bias in TBW_SFB7_ with reference to the isotope dilution method in athletes [8,9,10] and supports our hypothesis that the BIS algorithm using standard anthropometric coefficients causes an estimation error in some athletes. Arakawa et al. reported that the arm segment was significantly more developed than any other body segment in female wrestlers compared to elite athletes engaged in other sports [11]. Theoretically, hypertrophy of the arm relative to the trunk and the leg leads to alteration of K_B_. Thus, unique muscle development in wrestlers may have led to the difference between the actual and the postulated K_B_, which then resulted in the proportional bias in TBW_SFB7_. In wrestlers, overestimation in TBW_BMI_ was not found. Since the ratio of the muscle volume was greater in wrestlers than untrained subjects [12], it can be presumed that wrestlers were better aligned to Moissl’s equations made for Caucasian. Moreover, TBW_BMI_ did not show any systematic bias. TBW_BMI_ was originally made to adjust various anthropometric characteristics of healthy subjects and patients. However, our results support the notion that BMI correction contributes to bridging the gap between standard and actual anthropometric characteristics in wrestlers.

Our results show a small mean bias and no systematic bias in TBW_BMI_ and TBW_SFB7_ with reference to ${\mathrm{TBW}}_{D_{2}O}$ for wrestlers and untrained subjects, respectively. This finding shows that BIS can accurately estimate TBW of Japanese, at least at a population-mean level. However, the Bland–Altman analysis also showed few liters of the limit of agreement in TBW_SFB7_ and TBW_BMI_ with reference to ${\mathrm{TBW}}_{D_{2}O}\;$(Figure 2). The validity of BIS at the individual level was confirmed using CCC with factors of precision and accuracy in this study. CCC for TBW_SFB7_ and TBW_BMI_ in non-athletes were 0.896 and 0.853, which indicates poor strength of agreement, whereas, in athletes, these were 0.984 and 0.976, which indicates substantial strength of agreement [28]. Therefore, similar to previous studies [10,29,30], we presumed that BIS with de Lorenzo’s and Moissl’s equations are relatively less valid at the individual level.

In this study, we estimated body composition using BIS by using hydration in FFM, which has been reported in Japanese athletes and untrained subjects [5]. Our data showed that estimated body composition using BIS were similar to those calculated using Siri’s 3C model, if TBW was accurately assessed. This result means that FFM hydration reported in a previous study [5] was true for our subjects. In addition, BIS can be used in body composition assessment as a replacement for the isotope dilution method. Notably, the demonstration of a good accuracy in the assessment of TBW and body composition by BIS with Moissl’s equation in wrestlers is crucial because they try to maintain the weight and muscle volume. Moreover, several wrestlers undergo dehydration weight loss before competitions. Easy and accurate assessment of TBW and body composition by BIS may help athletes and coaches manage body conditions.

This study had some limitations. Although our data indicated that Moissl’s equation, which included correction in accordance with anthropometric characteristics, improved the TBW estimation only in wrestlers, the anthropometric characteristics of the athletes was more varied. Moreover, this study was conducted only in male subjects to ignore effects of menstrual edema on BIS accuracy. Therefore, additional studies are necessary to confirm the variability of BIS with Moissl’s equation in female athletes, athletes who have different anthropometric characteristics, and athletes of other races. Additionally, we determined the K_B_ value from a Japanese anthropometric database. However, we did not measure the actual K_B_ for each subject. We believe that this is one of the potential reasons why BIS has relatively less validity at the individual level. Future studies should be conducted to show that using the actual anthropometric coefficients improves the estimation error of BIS at the individual level in Japanese subjects.

## 5. Conclusions

BIS with de Lorenzo’s equation accurately estimates the TBW and body composition in male Japanese untrained subjects. However, a systematic bias is seen in male Japanese wrestlers. BIS with Moissl’s equation improves the systematic bias and provides accurate estimation of the TBW and body composition in wrestlers likely because BMI correction bridges the gap between the standard and actual anthropometric characteristics. The accurate assessment of TBW and body composition using BIS can facilitate the management of body conditions in wrestlers.

## Figures and Tables

**Table 1:** Anthropometric characteristics.

Variable	Unit	Untrained (*n* = 31)				Wrestlers (*n* = 18)			
Age	year	23	±	4	(20–37)	21	±	1 ^†^	(19–22)
Height	cm	173.4	±	6.5	(161.3–192.1)	167.8	±	3.7 ^†^	(160.4–173.4)
Weight	kg	63.9	±	6.2	(50.3–82.4)	72.2	±	8.3 ^††^	(62.9–91.6)
BMI	kg/m^2^	21.2	±	1.7	(18.0–25.1)	25.6	±	2.1 ^††^	(22.6–31.4)
D_b_	g/mL	1.065	±	0.009	(1.049–1.083)	1.074	±	0.011 ^†^	(1.037–1.086)

Values are mean ± SD (range). BMI, body mass index. D_b_, body density. ^†^ *p* < 0.05 and ^††^ *p* < 0.001 vs. untrained subjects.

**Table 2:** Body resistance and body water content.

Variable	Unit	Untrained (*n* = 31)			Wrestlers (*n* =18)		
R_e_	Ohm	642	±	71	506	±	45 ^††^
R_i_	Ohm	1226	±	213	852	±	111 ^††^
TBW_D2O_	Liter	38.1	±	3.8	46.0	±	4.5 ^††^
TBW_SFB7_	Liter	38.1	±	4.3	46.3	±	5.4 ^††^
ECW_SFB7_	Liter	16.0	±	1.9	18.6	±	2.1 ^††^
ICW _SFB7_	Liter	22.1	±	2.7	27.6	±	3.5 ^††^
TBW_BMI_	Liter	39.4	±	4.0 **	46.2	±	4.9 ^††^
ECW_BMI_	Liter	15.6	±	1.8	18.1	±	2.0 ^††^
ICW_BMI_	Liter	23.8	±	2.5	28.1	±	3.1 ^††^

Values are mean ± standard deviation. ** *p* < 0.001 vs. ${\mathrm{TBW}}_{D_{2}O}$ within group. ^††^ *p* < 0.001 vs. untrained subjects. R_e_, resistance of extracellular components. R_i_, resistance of intracellular components. TBW, total body water. ECW, extracellular water. ICW, intracellular water.

**Table 3:** Body composition estimated from total body water (TBW) measured using the D_2_O dilution technique and bioimpedance spectroscopy (BIS).

Variable	Unit	Untrained (*n* = 31)			Wrestlers (*n* = 18)		
FFM_3C_	kg	53.0	±	5.1	63.3	±	5.8 ^††^
%Fat_3C_	%	16.9	±	3.5	12.1	±	4.3 ^††^
FFM_SFB7_	kg	52.8	±	6.0	64.0	±	7.5 ^††^
%Fat_SFB7_	%	17.3	±	4.7	11.3	±	4.6 ^††^
FFM_BMI_	kg	54.6	±	5.6 **	63.9	±	6.7 ^††^
%Fat_BMI_	%	14.4	±	4.9 **	11.3	±	5.1 ^†^

Values are mean ± SD. ** *p* < 0.001 vs. 3C model within group. ^†^ *p* < 0.05 and ^††^ *p* < 0.001 vs. untrained subjects. FFM, fat-free mass. %Fat, % fat mass.

## References

[1] Kyle U.G., Bosaeus I., De Lorenzo A.D., Deurenberg P., Elia M., Gómez J.M., Heitmann B.L., Kent-Smith L., Melchior J.-C., Pirlich M. (2004). Bioelectrical impedance analysis--part I: Review of principles and methods. Clin. Nutr..

[2] van Marken Lichtenbelt W.D., Westerterp K.R., Wouters L., Luijendijk S.C. (1994). Validation of bioelectrical-impedance measurements as a method to estimate body-water compartments. Am. J. Clin. Nutr..

[3] Seoane F., Abtahi S., Abtahi F., Ellegard L., Johannsson G., Bosaeus I., Ward L.C. (2015). Mean expected error in prediction of total body water: A true accuracy comparison between bioimpedance spectroscopy and single frequency regression equations. Biomed. Res. Int..

[4] Cox-Reijven P.L., Soeters P.B. (2000). Validation of bio-impedance spectroscopy: Effects of degree of obesity and ways of calculating volumes from measured resistance values. Int. J. Obes. Relat. Metab. Disord..

[5] Sagayama H., Yamada Y., Ichikawa M., Kondo E., Yasukata J., Tanabe Y., Higaki Y., Takahashi H. (2020). Evaluation of fat-free mass hydration in athletes and non-athletes. Eur. J. Appl. Physiol..

[6] De Lorenzo A., Andreoli A., Matthie J., Withers P. (1997). Predicting body cell mass with bioimpedance by using theoretical methods: A technological review. J. Appl. Physiol..

[7] Moissl U.M., Wabel P., Chamney P.W., Bosaeus I., Levin N.W., Bosy-Westphal A., Korth O., Muller M.J., Ellegard L., Malmros V. (2006). Body fluid volume determination via body composition spectroscopy in health and disease. Physiol. Meas..

[8] Quiterio A.L., Silva A.M., Minderico C.S., Carnero E.A., Fields D.A., Sardinha L.B. (2009). Total body water measurements in adolescent athletes: A comparison of six field methods with deuterium dilution. J. Strength Cond. Res..

[9] Kerr A., Slater G., Byrne N., Chaseling J. (2015). Validation of bioelectrical impedance spectroscopy to measure total body water in resistance-trained males. Int. J. Sport Nutr. Exerc. Metab..

[10] Matias C.N., Judice P.B., Santos D.A., Magalhaes J.P., Minderico C.S., Fields D.A., Sardinha L.B., Silva A.M. (2016). Suitability of bioelectrical based methods to assess water compartments in recreational and elite athletes. J. Am. Coll. Nutr..

[11] Arakawa H., Yamashita D., Arimitsu T., Sakae K., Shimizu S. (2015). Anthropometric Characteristics of Elite Japanese Female Wrestlers. Int. J. Wrestl. Sci..

[12] Kanehisa H., Fukunaga T. (1999). Profiles of musculoskeletal development in limbs of college Olympic weightlifters and wrestlers. Eur. J. Appl. Physiol. Occup. Physiol..

[13] Yamada Y., Masuo Y., Yokoyama K., Hashii Y., Ando S., Okayama Y., Morimoto T., Kimura M., Oda S. (2009). Proximal electrode placement improves the estimation of body composition in obese and lean elderly during segmental bioelectrical impedance analysis. Eur. J. Appl. Physiol..

[14] Sagayama H., Yoshimura E., Yamada Y., Ichikawa M., Ebine N., Higaki Y., Kiyonaga A., Tanaka H. (2014). Effects of rapid weight loss and regain on body composition and energy expenditure. Appl. Physiol. Nutr. Metab..

[15] Yamada Y., Yokoyama K., Noriyasu R., Osaki T., Adachi T., Itoi A., Naito Y., Morimoto T., Kimura M., Oda S. (2009). Light-intensity activities are important for estimating physical activity energy expenditure using uniaxial and triaxial accelerometers. Eur. J. Appl. Physiol..

[16] Yamada Y., Hashii-Arishima Y., Yokoyama K., Itoi A., Adachi T., Kimura M. (2018). Validity of a triaxial accelerometer and simplified physical activity record in older adults aged 64-96 years: A doubly labeled water study. Eur. J. Appl. Physiol..

[17] Sagayama H., Jikumaru Y., Hirata A., Yamada Y., Yoshimura E., Ichikawa M., Hatamoto Y., Ebine N., Kiyonaga A., Tanaka H. (2014). Measurement of body composition in response to a short period of overfeeding. J. Physiol. Anthropol..

[18] Cole K.S., Cole R.H. (1941). Dispersion and absorption in dielectrics I. Alternating current characteristics. J. Chem. Phys..

[19] Yamada Y., Watanabe Y., Ikenaga M., Yokoyama K., Yoshida T., Morimoto T., Kimura M. (2013). Comparison of single- or multifrequency bioelectrical impedance analysis and spectroscopy for assessment of appendicular skeletal muscle in the elderly. J. Appl. Physiol..

[20] Shiose K., Yamada Y., Motonaga K., Sagayama H., Higaki Y., Tanaka H., Takahashi H. (2016). Segmental extracellular and intracellular water distribution and muscle glycogen after 72-h carbohydrate loading using spectroscopic techniques. J. Appl. Physiol..

[21] Kouchi M., Mochimaru M. (2005). AIST Anthropometric Database. https://www.airc.aist.go.jp/dhrt/91-92/index.html.

[22] Siri W.E., Brozek J., Henschel A. (1961). Body composition from fluid spaces and density: Analysis of methods. Techniques for Measuring Body Composition.

[23] Lin L.I. (1989). A concordance correlation coefficient to evaluate reproducibility. Biometrics.

[24] Moon J.R., Tobkin S.E., Roberts M.D., Dalbo V.J., Kerksick C.M., Bemben M.G., Cramer J.T., Stout J.R. (2008). Total body water estimations in healthy men and women using bioimpedance spectroscopy: A deuterium oxide comparison. Nutr. Metab..

[25] Gordon C.C., Churchill T., Clauser C.E., Bradtmiller B., McConville J.T., Tebbetts I., Walker R.A. (1989). Anthropometric Survey of US Army Personnel: Summary Statistics, Interim Report for 1988.

[26] Wulan S.N., Westerterp K.R., Plasqui G. (2010). Ethnic differences in body composition and the associated metabolic profile: A comparative study between Asians and Caucasians. Maturitas.

[27] Rush E.C., Freitas I., Plank L.D. (2009). Body size, body composition and fat distribution: Comparative analysis of European, Maori, Pacific Island and Asian Indian adults. Br. J. Nutr..

[28] McBride G. (2005). A Proposal for Strength-of-Agreement Criteria for Lin’s Concordance Correlation Coefficient.

[29] Matias C.N., Santos D.A., Gonçalves E.M., Fields D.A., Sardinha L.B., Silva A.M. (2013). Is bioelectrical impedance spectroscopy accurate in estimating total body water and its compartments in elite athletes?. Ann. Hum. Biol..

[30] Matias C., Noujeimi F., Sardinha L., Teixeira V., Silva A. (2018). Total body water and water compartments assessment in athletes: Validity of multi-frequency bioelectrical impedance. Sci. Sports.

